# Impact of HIV and Type 2 diabetes on Gut Microbiota Diversity, Tryptophan Catabolism and Endothelial Dysfunction

**DOI:** 10.1038/s41598-018-25168-3

**Published:** 2018-04-30

**Authors:** Hedda Hoel, Malene Hove-Skovsgaard, Johannes R. Hov, Julie Christine Gaardbo, Kristian Holm, Martin Kummen, Knut Rudi, Felix Nwosu, Jørgen Valeur, Marco Gelpi, Ingebjørg Seljeflot, Per Magne Ueland, Jan Gerstoft, Henrik Ullum, Pål Aukrust, Susanne Dam Nielsen, Marius Trøseid

**Affiliations:** 1 Research Institute of Internal Medicine, Oslo University Hospital, Rikshospitalet, 0424 Oslo, Norway; 2 K.G. Jebsen Inflammation Research Centre, University of Oslo, 0424 Oslo, Norway; 3 Institute of Clinical Medicine, University of Oslo, 0424 Oslo, Norway; 4 Department of Microbiology, Oslo University Hospital Rikshospitalet, 0424 Oslo, Norway; 5 Section of Clinical Immunology and Infectious Diseases, Oslo University Hospital, Rikshospitalet, 0424 Oslo Norway; 6 Department of Infectious Diseases, Rigshospitalet, University of Copenhagen, 2100 Copenhagen, Denmark; 7 Viro-immunology Research Group, Department of Infectious Diseases, Rigshospitalet, University of Copenhagen, 2100 Copenhagen, Denmark; 8 Norwegian PSC Research Center, Department of Transplantation Medicine Oslo University Hospital Rikshospitalet, 0424 Oslo, Norway; 9 Section of Gastroenterology, Department of Transplantation Medicine, Oslo University Hospital Rikshospitalet, 0424 Oslo, Norway; 10 Department of Chemistry, Biotechnology and Food Science, Norwegian University of Life Sciences, 1430 Ås, Norway; 11 Unger-Vetlesen Institute, Lovisenberg Diaconal Hospital, 0440 Oslo, Norway; 12 Centre for Clinical Heart Research, Department of Cardiology, Oslo University Hospital Ullevål, Oslo, Norway; 13 Faculty of Medicine, University of Oslo, 0424 Oslo, Norway; 14 Section for Pharmacology, Department of Clinical Science, University of Bergen, 5007 Bergen, Norway; 15 Department of Clinical Immunology, Rigshospitalet, Copenhagen University Hospital, 2100 Copenhagen, Denmark

## Abstract

HIV infection and type 2 diabetes are associated with altered gut microbiota, chronic inflammation, and increased cardiovascular risk. We aimed to investigate the combined effect of these diseases on gut microbiota composition and related metabolites, and a potential relation to endothelial dysfunction in individuals with HIV-infection only (n = 23), diabetes only (n = 16) or both conditions (n = 21), as well as controls (n = 24). Fecal microbiota was analyzed by Illumina sequencing of the 16 S rRNA gene. Markers of endothelial dysfunction (asymmetric dimethylarginine [ADMA]), tryptophan catabolism (kynurenine/tryptophan [KT]-ratio), and inflammation (neopterin) were measured by liquid chromatography-tandem mass spectrometry. The combination of HIV and type 2 diabetes was associated with reduced gut microbiota diversity, increased plasma KT-ratio and neopterin. Microbial genes related to tryptophan metabolism correlated with KT-ratio and low alpha diversity, in particular in HIV-infected with T2D. In multivariate analyses, KT-ratio associated with ADMA (β = 4.58 [95% CI 2.53–6.63], p < 0.001), whereas microbiota composition per se was not associated with endothelial dysfunction. Our results indicate that tryptophan catabolism may be related to endothelial dysfunction, with a potentially detrimental interaction between HIV and diabetes. The potential contribution of gut microbiota and the impact for cardiovascular risk should be further explored in prospective studies powered for clinical end points.

## Introduction

HIV affects more than 35 million people worldwide, and with increasing use of antiretroviral treatment (ART) life expectancy has increased substantially^1^. In an ageing HIV-infected population, the relative burden of non-AIDS comorbidities such as cardiovascular and related metabolic disorders is increasing^2^. In addition to traditional risk factors such as hyperlipidemia and smoking, chronic inflammation may contribute to increased risk and progression of cardiovascular and metabolic comorbidities^3^.

Type 2 diabetes (T2D) is one of the most common chronic diseases worldwide, and like HIV characterized by chronic inflammation, atherosclerosis and increased cardiovascular risk^4^. Although it is debated if the risk of T2D is higher in HIV-infected patients than in the general population^5,6^, an ageing HIV-infected population in combination with a worldwide emerging diabetes epidemic is rapidly becoming a clinical challenge requiring increased attention and knowledge about the potential interacting effect of these two chronic diseases.

Endothelial dysfunction occurs in early stages of the atherosclerotic process and is also an early feature of T2D^4^. Asymmetric dimethylarginine (ADMA) is a well-established biomarker for nitric oxide (NO)-dependent endothelial dysfunction in both HIV and T2D^7–9^. We have recently shown an additive effect of HIV and T2D on endothelial dysfunction as measured by ADMA^10^. Previous works have suggested a potential role of inflammation in this patient group, with low levels of IL-10 production in HIV-infected with diabetes^11^, and soluble TNFR1 predicting incident diabetes in HIV-infected on ART^12^, but the potential triggers remain unknown.

A healthy gut microbiota contains a highly diverse population of bacteria which is involved in the regulation of numerous metabolic and inflammatory pathways. The gut microbiota exerts its functions not only in the gut, but also systemically, and is altered in metabolic and cardiovascular diseases such as obesity, T2D and atherosclerosis^13–15^. During the last years, an altered gut microbiome has also been well documented in HIV infection. Of note, the gut microbiota changes observed in several HIV-infected cohorts^16–19^ partly overlap with those found in patients with obesity and T2D, with reduced biodiversity and lower relative abundance of commensal microbes, including butyrate-producing bacteria^13,15,20^. There is, however, a great variability in the findings, partly due to variations in sampling and sequencing methods as well as ethnicity, diet, age, geography, ART and modes of transmission, including sexual practice^16–19,21–26^. Whereas reduced alpha diversity is found in several cohorts of untreated HIV infection, and partly relates to low CD4 count, studies have been conflicting regarding alpha diversity in ART-treated cohorts^18,21,23,26^. Hence, a specific HIV-associated dysbiosis remains to be defined.

A particular finding reported in HIV-infected cohorts is that microbiota alterations have been associated with tryptophan catabolism and systemic inflammation^19,26^. Tryptophan is an essential amino acid, and is mainly catabolized through the kynurenine pathway to metabolites collectively called kynurenines. The first and rate-limiting step in the kynurenine pathway is the oxidative cleavage of the indole ring of tryptophan to form N-formyl-kynurenine, which is spontaneously decomposed to kynurenine. This step is catalysed by indolamine 2,3-dioxygenase-1 (IDO-1), which is induced by interferon (IFN)-γ^19^. Altered tryptophan metabolism has been linked to immune activation and increased mortality in HIV-infected individuals^27,28^, and has been associated with increased cardiovascular mortality in the general population^29,30^. Recently, tryptophan catabolism through the kynurenine pathway was even shown to be predictive of incident T2D in individuals with coronary artery disease^31^.

Disturbed gut microbiota composition, IFN-γ-induced tryptophan catabolism and endothelial dysfunction could therefore represent pathogenic pathways potentially interacting in HIV infection and T2D. We set out to investigate this hypothesis in a cohort of individuals with HIV-infection only, T2D only or both condition, as well as controls.

## Research Design and Methods

### Study design and participants

The total study cohort consisted of 100 participants in a cross-sectional study^10^. Gut microbiota samples were available from 84 participants who were included in the present study: 21 HIV-infected individuals with T2D (HIV+T2D), 23 HIV-infected without T2D (HIV only), 16 individuals with T2D without HIV (T2D only) and 24 individuals without HIV and T2D (denoted controls). Study participants were included from Departments of Infectious Diseases, University Hospital of Copenhagen, Rigshospitalet and Hvidovre Hospital, from Department of Endocrinology, and Center of Inflammation and Metabolism, University Hospital of Copenhagen, Rigshospitalet. Exclusion criteria were immunosuppressive treatment, acute infections, malignancy, and pregnancy.

All HIV-infected individuals were on stable ART for at least 11 months, with the exception of five patients who participated in a switch study from abacavir/lamivudine to emtricitabine/tenofovir disoproxil fumarate. Time on ART did not differ significantly between the HIV only group and the HIV+ T2D group. With the exception of three episodes with blips (<200 copies/mL), all individuals remained virally suppressed (i.e., <40 copies/mL) during time on ART. All persons with T2D were treated with diet and/or oral anti-diabetics and/or insulin, and had a normal fasting glucose (<6.1 mmol/L) and HbA1c <6.2% (44 mmol/mol). The proportion of individuals receiving oral anti-diabetics and insulin did not differ significantly between the T2D only group and the HIV +T2D group (Table 1).

**Table 1:** Demographic and biochemical characteristics of the study population.

	Controls (n = 24)	Type 2 diabetes (n = 16)	HIV (n = 23)	HIV + type 2 diabetes (n = 21)	P
Age (years)	57 (54–60)	57 (54–60)	54 (51–58)	57 (54–60)	0.512
Gender (% male)	92	69	96	90	0.061
Smokers (%)	79	88	78	57	0.154
Use of medication (%)					
PI	—	—	65^d^	33	0.035
NNRTI	—	—	35^d^	71	0.015
Statins	12^b,d^	69	4^b,d^	71	< 0.001
Betablockers	0^d^	6^d^	5^d^	29	0.006
ACE inhibitor/ATII antagonist	21^d^	40	9^b,d^	62	0.007
Oral antidiabetics	—	75	—	81	0.663
Insulin	—	13	—	24	0.384
Hypertension (%)	29^d^	48^d^	13^b,d^	81	<0.001
HIV transmission (MSM, heterosexual, IDU) (%)	—	—	87/3/0	62/15/23	0.131
Time on stable ART (months)	—	—	22.5 (12.4–32.6)	25.4 (13.2–37.7)	0.693
Physical activity ( < 1/1–2/ ≥ 3 times/week)	17/38/45^d^	31/31/38	41/27/32	45/35/20	0.023
CD4 count (cells/μL)	861 (721–1000)^c,d^	1131 (921–1342)^c,d^	612 (490–734)	646 (511–780)	<0.001
LDL cholesterol (mmol/L)	3.5 (3.2–3.8)^b,d^	2.3 (1.8–2.8)	3.4 (3.0–3.7)^b,d^	2.4 (2.0–2.8)	<0.001
HbA1c (mmol/mol)	37 (36–38)^b,d^	57 (51–62)	35 (34–37)^b,d^	48 (44–52)	<0.001
HbA1c (%)	5.5 (5.4–5.6)	7.4 (6.8–7.8)	5.4 (5.3–5.5)	6.5 (6.2–6.9)	
BMI (kg/m^2^)	25 (24–26)	28(26–30)	25 (23–27)	26 (23–28)	0.064
Triglycerides (mmol/L)	1.4 (1.0–1.9)^d^	2.1 (1.5–2.8)	1.9 (1.3–2.4)	2.7 (1.9–3.5)	0.026
HDL cholesterol (mmol/L)	1.6 (1.4–1.8)^b,d^	1.3 (1.1–1.5)	1.4 (1.2–1.5)	1.2 (1.0–1.4)	0.010
Systolic BP (mmHg)	135 (129–141)	136 (130–142)	128 (122–134)	132 (123–140)	0.322
ADMA (µmol/L)	0.55 (0.52–0.58)^c,d^	0.60 (0.54–0.65)	0.60 (0.56–0.63)^d^	0.67 (0.62–0.72)	0.001

P-value refers to one-way ANOVA for continuous data and Chi-Square or Fisher’s exact test for categorical data. Results are given as % or mean and 95% CI. ^b,c,d^ Refers to t-test; ^b^p < 0.05 vs. T2D, ^c^p < 0.05 vs. HIV, ^d^p < 0.05 vs. HIV + T2D. LDL: low density lipoprotein, HDL: high density lipoprotein, BMI: body mass index, BP: blood pressure, ADMA: Asymmetric dimethylarginine, MSM: men who have sex with men, IDU: intravenous drug use.

Apparently healthy controls, based on medical history and clinical examination, were recruited among hospital staff. The study was performed in accordance with the declaration of Helsinki, approved by the local ethical committee (H-4-2012-076 CIM VEK) and the Danish Data Protection Agency, and written informed consent was obtained from all subjects.

### Biochemistry and blood sampling

Fasting blood samples were collected, and glucose, HbA1c, lipid parameters, CD4 T cell count and HIV RNA were measured as routine analyses. To assess endothelial dysfunction, L-arginine and ADMA were measured in snap-frozen EDTA-plasma by high performance liquid chromatography (HPLC) and precolumn derivatization with *o*-phthaldialdehyde (Sigma Chemicals Co, St.Louis, MO, USA) as described^10^. Inter- and intra-assay CV’s were <5%. Plasma levels of neopterin, kynurenine and tryptophan were analyzed at BEVITAL (www.bevital.no) by liquid chromatography–tandem mass spectrometry (LC-MS/MS) as previously described^32^. Kynurenine/tryptophan (KT)-ratio was used as a measure of IDO-1 activation^19^, and plasma levels of neopterin as a measure of IFN-γ activity^33–36^.

### DNA extraction from stool samples and 16 s rRNA sequencing of gut microbiota

Two stool samples without preservatives were collected from each participant, shipped by mail to Rigshospitalet and stored at −80 °C until further use. The samples were processed and DNA was extracted as previously described^18^. Extracted DNA was PCR amplified targeting the 16 S rRNA gene using universal primers (for all prokaryotic organisms) flanking V3-V4 region of 16 S gene modified with addition of TruSeq Illumina adapters. PCR amplification consisted of 30 cycles of 95 °C for 30 seconds, 50 °C for 30 seconds, 75 °C for 45 seconds and followed by a final elongation at 72 °C for 7 minutes. Each PCR-amplicon was quantified using Quant-iT^TM^ PicoGreen® dsDNA kit (Life Technologies, USA), then normalized and pooled in equal concentrations. The pooled amplicons were purified with E.N.Z.A. Cycle Pure Kit (Omega Bio-Tek, Norcross, Georgia, USA) and eluted in 0.1 mM Tris buffer (pH 8.5). After E.N.Z.A. kit purification, there was a final DNA quantification of the amplicon pool. Sequencing was performed on the Illumina MiSeq platform using the 250 base pair, paired end protocol (Illumina, San Diego, California, USA) at Norwegian Sequencing Centre (Oslo, Norway).

#### Fecal calprotectin

Fecal samples were extracted by using Calex Cap Device (Bühlmann Laboratories AG, Schönenbuch, Switzerland), and calprotectin concentrations were analyzed in the extracts by using an enzyme-linked immunoassay test (Bühlmann fCAL ELISA, Bühlmann Laboratories AG, Schönenbuch, Switzerland), according to the manufacturer’s instructions. All extracts were analyzed in duplicates and fecal calprotectin levels were reported as mean values of the duplicates (mg/kg). As a large proportion of samples were below the lower limit of detection (40 mg/kg), the values were categorized as non-detectable, normal or elevated (>100 mg/kg).

### Sequence analysis and bioinformatics

Paired-end reads were merged with FLASH (Fast length adjustment of short reads to improve genome assemblies) version 1.2.11^37^. The merged reads were de-multiplexed, pre-processed and subsequently analyzed with QIIME (Quantitative Insights Into Microbial Ecology) version 1.8.0 software package^38^. Taxonomy classification was performed using “closed-reference OTU picking” against the Greengenes version 13.8 OTU database with 97% cluster identity. For each individual, we used the sample with the highest read count after pre-processing. Alpha diversity was calculated using number of observed bacterial species and Shannon diversity index as previously described^18^. To assess the functional characteristics of gut microbiota alterations, we performed a PICRUSt (Phylogenetic Investigation of Communities by Reconstruction of Unobserved States) analysis^39^, normalized to 5,337 reads per sample.

### Statistics

Continuous data were analyzed using one-way ANOVA for comparison of multiple groups, followed by t test for between group comparisons, with the exception of relative abundance of bacterial species that were not normally distributed. Hence, relative abundance of bacterial species was compared with Kruskal-Wallis test for comparison of multiple groups, followed by Mann Whitney U test for between group comparisons. Categorical data were compared with Chi squared test. Correlations were performed with Pearson correlation, and only statistically significant correlations (p < 0.05) are reported. False-discovery rate (FDR) was calculated according to Benjamini-Hochberg, and denoted Q_FDR_. Potential predictors of endothelial dysfunction were further investigated in a stepwise linear regression analysis. Skewed data were log-transformed before Pearson correlations and linear regression analyses. The statistical analyses were performed with SPSS software, version 21.0 (IBM SPSS Statistics), and FDR-values were calculated in R (v3.4).

### Data availability

The datasets generated during and/or analyzed during the current study are not publicly available due to Norwegian legislation about general data protection regulation, but are available from the corresponding author on reasonable request.

## Results

### Baseline characteristics

Baseline characteristics are given in Table 1. There was more use of ACE-inhibitors, angiotensin 2 blockers and statins in patients with T2D irrespective of HIV-status, whereas use of beta-blockers was more prevalent in HIV-infected patients with T2D. HIV-infected patients with T2D were more often treated with non-nucleoside reverse transcriptase inhibitors (NNRTIs) and less often with protease inhibitors (PIs) compared to HIV-infected patients without T2D. Those with combined T2D and HIV infection had the highest levels of triglycerides and the lowest levels of high-density lipoprotein (HDL) cholesterol, but there was no difference in HbA1c in patients with T2D according to HIV status. Furthermore, low-density lipoprotein (LDL) cholesterol was lower in patients with T2D irrespective of HIV status, probably reflecting higher use of statins. Finally, there was a higher proportion of sedentary individuals (exercising <once/week) in the HIV+T2D group compared to controls.

### Impact of HIV, T2D and both on endothelial dysfunction, tryptophan metabolism, and inflammation

**Figure 1:**
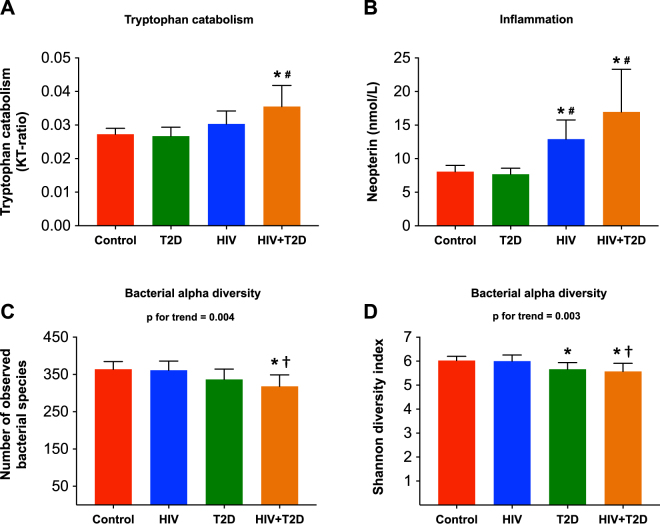
The impact of HIV, T2D and both (HIV + T2D) on (**A**) tryptophan catabolism (KT-ratio), (**B**) inflammation (neopterin), (**C** and **D**) gut microbiota diversity (number of observed bacterial species and Shannon diversity index). Controls (red), HIV only (blue), T2D only (green) and HIV-infected with T2D (orange). *p < 0.05 vs. controls, ^#^p < 0.05 vs. T2D only, ^†^p < 0.05 vs. HIV only.

As previously described, HIV-infected patients with T2D had higher concentration of ADMA as a marker of endothelial dysfunction compared with controls and HIV-infected patients without T2D^10^ (Table 1). In addition, HIV-infected patients had higher plasma KT-ratio as a marker of increased IDO-1 induced tryptophan metabolism and higher levels of the pro-inflammatory marker neopterin, with the highest levels in those with accompanying T2D (Fig. 1A,B). In contrast, T2D alone was not associated with increased KT-ratio or neopterin levels.

### HIV-infected patients with T2D have an altered gut microbiota with reduced alpha diversity

As depicted in Fig. 1C and D, the lowest alpha diversity (number of observed bacterial species) was found in HIV-infected patients with T2D, followed by T2D alone, HIV alone, and healthy controls. Of note, there was no significant difference between HIV alone and healthy controls whereas HIV + T2D had a significantly lower number of observed bacterial species compared to both HIV alone and controls. The same pattern was seen when applying the Shannon diversity index, with the lowest alpha diversity in HIV + T2D, significantly lower than HIV alone and controls (p for trend = 0.003).

### Diabetes treatment, Framingham score and mode of HIV transmission are associated with alpha diversity

Factors associated with alpha diversity measures are given in Table 2, including physical activity and HDL cholesterol which were associated with higher alpha diversity measures, and smoking and Framingham risk score being associated with lower alpha diversity. With respect to diabetes treatment, metformin was associated with higher alpha diversity, as previously reported^13^. Concerning HIV transmission, there was a higher alpha diversity in men who have sex with men (MSM), also in line with a recent report^23^. In contrast, BMI, time on ART, CD4+ T cell count, type of ART (PI/NNRTI) or use of statins were not associated with alpha diversity measures (data not shown).

**Table 2:** Association between covariates and gut microbiota diversity.

	Observed bacterial species		p	Q_FDR_	Shannon diversity Index		p	Q_FDR_
MSM (yes/no)	363(339–386)	321 (290–351)	0.030	0.054	6.06 (5.85–6.27)	5.56 (5.21–6.90)	0.015	0.038
Physical active (< 1/1–2/week)	355 (332–378)	332 (309–356)	0.151	0.164	5.91 (5.69–6.14)	5.63 (5.36–5.89)	0.038	0.057
Insulin (yes/no)	256 (204–308)	355 (343–367)	<0.001	<0.001	4.69 (4.21–5.18)	5.94 (5.83–6.05)	<0.001	<0.001
Metformin (yes/no)	336 (313–359)	296 (255–339)	0.066	0.099	5.75 (5.55–6.00)	5.21 (4.80–5.64)	0.017	0.051
Betablocker (yes/no)	300 (240–359)	351 (339–364)	0.017	0.038	5.51 (4.94–6.09)	5.87 (5.74–6.01)	0.110	0.110
Statin (yes/no)	332 (309–355)	355 (339–370)	0.094	0.121	5.69 (5.45–5.92)	5.92 (5.76–6.08)	0.089	0.110
Smoking (yes/no)	317 (288–346)	356 (343–370)	0.007	0.021	5.58 (5.27–5.89)	5.92 (5.78–6.02)	0.023	0.052
HDL cholesterol	r = 0.20		0.066	0.099	r = 0.24		0.030	0.054
Framingham 10 year CVD risk	r = −0.49		<0.001	<0.001	r = −0.42		<0.001	<0.001

Covariates with p < 0.10 are given. Data as mean (95% CI). Smoking and HDL cholesterol are part of the Framingham 10 year CVD risk, and were not entered as separate covariates in the multivariate linear regression model. MSM: men who have sex with men. MSM status was only available in HIV infected individuals. MSM status “no” refers to heterosexual transmission, IDU and unknown transmission.

### HIV-infected patients with T2D have altered gut microbiota composition and increased fecal calprotectin levels

Looking at microbiota composition in more detail, no significant differences were observed between the groups among the major bacterial phyla. Significantly different taxa on order and genus level are summarized in Supplementary Table S1 showing differences in HIV + T2D as compared with the other groups. On order level, there was an expansion of Enterobacteriales and Lactobacillales in the HIV + T2D group, the latter being driven by an increase in *Streptococcus* on genus levels. Among the more abundant taxa on the genus level, microbes from the family of Lachnospiracea (*Lachnospira*, *Lachnobacterium, Anaerostipes*) were depleted in the HIV + T2D group. Although the Lachnospiracea family is known for its capacity to produce butyrate, the overall capacity for butyrate metabolism as predicted by a PICRUSt analysis, did not differ between the groups (p = 0.70).

Of note, none of the bacterial taxa were significantly different between the groups after FDR adjustment, and differences in individual bacterial taxa should therefore be interpreted with caution. In order to capture the overall local inflammation in the gut, we measured fecal calprotectin levels, finding a higher fraction of individuals with elevated calprotectin levels (>100 mg/kg feces) in HIV + T2D (43%) compared to the other groups (HIV only 22%, T2D 13%, controls 17%, p for trend = 0.048). However, log-transformed fecal calprotectin levels did not correlate with soluble markers, including ADMA (r = 0.11, p = 0.308), L-arginin/ADMA ratio (r = −0.09, p = 0.439), KT-ratio (r = 0.12, p = 0.281), neopterin (r = 0.11, p = 0.307) or log-transformed C-reactive protein (CRP) levels (r = 0.09, p = 0.440).

### Tryptophan catabolizing bacteria are associated with KT-ratio and plasma neopterin but not with endothelial dysfunction

We next examined the potential impact of tryptophan metabolizing microbes in the gut microbiota by performing a PICRUSt analysis. Bacterial taxa contributing to tryptophan metabolism belonged mainly to the phylum Proteobacteria, including *Burkholderia*, *Pseudomonas*, and *Bacillus*, as previously reported^19^ (Supplementary Table S2). Bacterial genes related to tryptophan metabolism correlated with KT-ratio and neopterin in the total population (r = 0.33, p = 0.002 and r = 0.38, p < 0.001) and particularly in HIV-infected patients with T2D (r = 0.52, p = 0.015 and r = 0.57, p = 0.007). Furthermore, tryptophan metabolizing microbes correlated negatively with gut microbiota diversity and again, these correlations were stronger in HIV-infected with T2D (Table 3).

**Table 3:** Correlations between alpha diversity measures, endothelial dysfunction and bacterial genes related to tryptophan metabolism, KT-ratio and inflammation in the total study population (n = 84) and the HIV-infected individuals with type 2 diabetes (n = 21).

Variable	Gut microbiota diversity				Endothelial dysfunction			
	Observed species		Shannon Index		L-Arginine/ADMA		ADMA	
	Total population	HIV + type 2 diabetes	Total population	HIV + type 2 diabetes	Total population	HIV + type 2 diabetes	Total population	HIV + type 2 diabetes
Bacterial tryptophan metabolism	r = −0.29, p = 0.007	**r = −0.54, p = 0.012**	r = −0.47, p < 0.001	**r = −0.73, p < 0.001**	r = −0.03, p = 0.824	r = −0.40, p = 0.083	r = 0.12, p = 0.267	r = 0.29, p = 0.222
KT-ratio	r = −0.25, p = 0.024	r = −0.28, p = 0.216	r = −0.22, p = 0.012	r = −0.25, p = 0.824	r = −0.24, p = 0.029	**r = −0.60, p = 0.006**	r = 0.45, p < 0.001	**r = 0.56**, **p = 0.010**
Neopterin	r = −0.29, p = 0.008	r = −0.40, p = 0.074	r = −0.25, p = 0.020	r = −0.31, p = 0.173	r = −0.27, p = 0.014	r = −0.40, p = 0.079	r = 0.43, p < 0.001	r = 0.47, p = 0.037
CRP	r = −0.30, p = 0.009	r = −0.32, p = 0.166	r = −0.29, p = 0.014	r = −0.35, p = 0.135	r = −0.21, p = 0.075	r = −0.20, p = 0.409	r = 0.15, p = 0.209	r = 0.30, p = 0.209

Data as Pearson correlations. Pearson correlations > 0.5 in bold. ADMA: Asymmetric dimethylarginine, CRP: C-reactive protein, KT: Kynurenin/Tryptophan. CRP-levels were log-transformed before correlation analyses.

Among the factors potentially confounding alpha diversity (as shown in Table 2), MSM status was associated with lower predicted abundance of tryptophan metabolizing microbes (median 4609 [IQR 897] vs. 5473 [IQR 499], p = 0.002). Of note, these factors, including MSM status, were not associated with KT-ratio. Tryptophan metabolizing microbes, although being correlated with KT-ratio and neopterin, did not correlate with endothelial dysfunction (Table 3). CRP levels correlated inversely with alpha diversity measures but not with markers of endothelial dysfunction (Table 3).

### Increased tryptophan catabolism is associated with endothelial dysfunction in multivariate analyses

As shown in Table 3, KT-ratio and neopterin were positively correlated with ADMA and negatively correlated with L-arginine/ADMA-ratio, suggesting an association between systemic inflammation, altered tryptophan metabolisms and endothelial dysfunction. Notably, these correlations were stronger in HIV-infected with T2D (Table 3).

**Figure 2:**
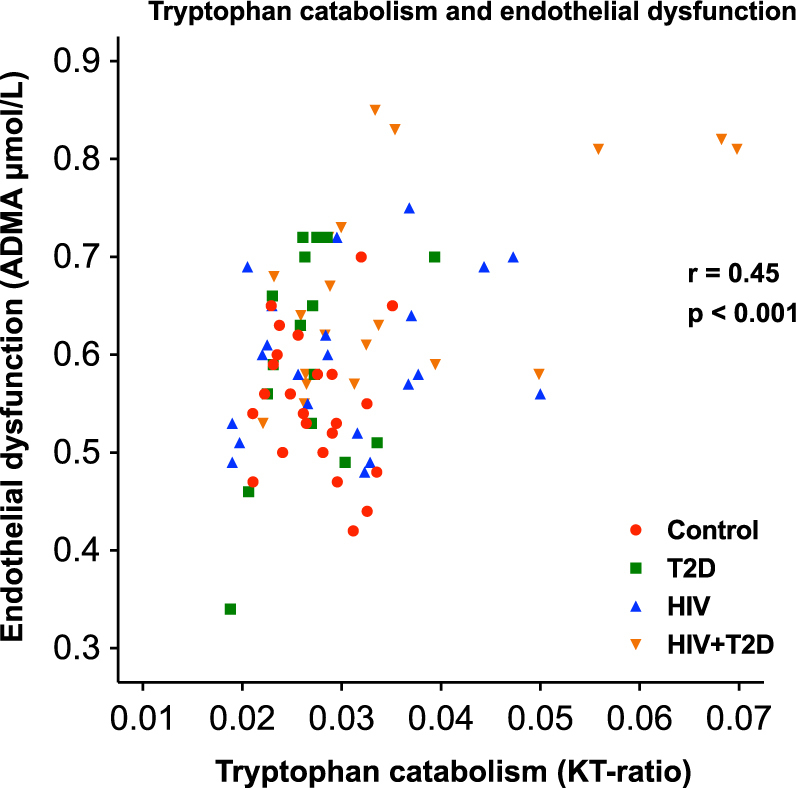
Association (Pearson correlation) between tryptophan catabolism (KT-ratio) and endothelial dysfunction assessed by ADMA in the total study population; controls (red), HIV only (blue), T2D only (green) and HIV-infected with T2D (orange).

To further investigate potential predictors of endothelial dysfunction, we performed a stepwise multivariate linear regression analysis with ADMA as dependent variable, and KT-ratio, group, traditional cardiovascular risk factors, gut microbiota diversity and factors affecting the gut microbiota (Table 2) as covariates. KT-ratio, log-transformed CRP, group, observed bacterial species, physical activity, diabetes treatment (insulin, oral), mode of HIV transmission (MSM, others), and Framingham 10 year CVD score were included in the multivariate model. Neopterin was excluded from the model due to the close correlation with KT-ratio (r = 0.78, p < 0.001). KT-ratio was associated with ADMA in multivariate analyses (Fig. 2). Hence, higher KT-ratio was associated with higher ADMA reflecting increased endothelial dysfunction (β = 4.58 [95% CI 2.53–6.63], p < 0.001) also after adjusting for confounders.

## Discussion

The main findings in the present study can be summarized as follows: i) The combination of HIV and T2D was associated with lower gut microbiota diversity, increased tryptophan catabolism, and higher levels of the pro-inflammatory marker neopterin, ii) gut microbiota diversity was negatively associated with smoking and Framingham score, and positively associated with metformin treatment, physical activity and mode of HIV transmission (MSM), iii) microbes encoding genes related to tryptophan metabolism were associated with KT-ratio and neopterin but not with endothelial dysfunction, whereas iv) tryptophan catabolism as measured by KT-ratio was associated with markers of endothelial dysfunction in multivariate analyses.

Our findings suggest that the combination of HIV and T2D have detrimental effects on systemic inflammation and tryptophan catabolism, which potentially may promote endothelial dysfunction. Progressive HIV-infection is characterized by chronic inflammation and IDO-1 activation, and increased KT-ratio has been associated with non-AIDS defining morbidity in HIV-infected US subjects on suppressive ART^40^. Interestingly, a study showed that greater decline in KT-ratio after 6 months of ART predicted a lower carotid intima media thickness in Ugandan individuals receiving ART^41^. The present study is, however, to the best of our knowledge, the first to link tryptophan catabolism to markers of endothelial dysfunction, and notably, these associations were particularly strong in subjects with both HIV infection and T2D.

Approximately 95% of dietary tryptophan is catabolized to kynurenine, which has immunosuppressive properties, partly by inhibiting T-cell proliferation and depleting Th17 cells, which in turn disfavours the mucosal barrier, thereby promoting low grade endotoxemia and T cell activation^42^. Activated T cells produce IFN-γ, which in turn activates tryptophan catabolism through upregulation of IDO-1, neopterin is almost exclusively released from IFN-γ activated monocytes/macrophages^33^, and plasma neopterin levels are associated with progressive HIV infection^34–36^.

Whereas plasma kynurenines and IFN-γ mediated immune activation have been associated with increased cardiovascular mortality in the general population^29,30^, and the metabolites of the kynurenine pathway may act as biomarkers for atherosclerotic disease, the mechanistic impact of activation of these pathways is not completely understood. Thus, whereas downstream metabolites of kynurenine seem to accelerate endothelial apoptosis and dysfunction *in vivo*^43^, and IDO-1 activation appears to promote atherosclerosis in mice through reduced IL-10 expression^44^, Zhang *et al*. identified atheroprotective effects of 3-HAA, a downstream tryptophan metabolite^45^. Also, systemic IDO-1 inhibition by 1-methyl-tryptophan resulted in increased atherosclerosis in mice^46^, implying that upregulation of IDO-1 may play a protective role against atherosclerosis. Nonetheless, our results suggest that IFN-γ activity and tryptophan catabolism may be closely linked to endothelial dysfunction, in particular in HIV-infected with T2D. Moreover, a recently published study showed that kynurenic acid-to-tryptophan ratio was associated with progression of carotid artery atherosclerosis in well-treated HIV patients^47^. However, if these associations represent harmful or counteracting beneficial effects of these metabolites will need to be further clarified.

HIV-associated alterations of the gut microbiota have been associated with systemic inflammation and tryptophan catabolism^19,26^. Our results confirm these findings, and even suggest that the association between tryptophan metabolising microbes and KT-ratio may be stronger in HIV-infected patients with T2D. Of note, the abundance of tryptophan metabolizing microbes was lower in MSM, which to our knowledge is a novel observation. Given the large diversity of the present study population with respect to mode of HIV transmission, comorbidities and medication, it is not possible to predict the potential contribution from the gut microbiota on tryptophan catabolism measured by KT-ratio. Nevertheless, KT-ratio was not affected by MSM-status or other potential confounders of gut microbiota composition.

In the present study, we found the largest gut microbiota alterations with low alpha diversity in HIV-infected patients with T2D, but several factors beyond the disease states could have influenced our findings. First, studies have been conflicting regarding alpha diversity in HIV-infected on ART, with some but not all studies reporting reduced diversity^18,21,23^, and in the present study, we found a reduced diversity only in HIV-infected with T2D. Second, recent studies have implicated MSM status as a potential confounder, being associated with higher, not lower alpha diversity^23,48^, in line with higher diversity among MSM in the present study. Third, metformin treatment was associated with higher diversity in line with a recent publication suggesting that the microbiota mediates some of the therapeutic effects of metformin through short-chain fatty acid production^13^. Finally, physical activity was associated with higher diversity and smoking with a lower diversity of the gut microbiota, in line with previous studies reporting higher bacterial diversity in individuals with cardiorespiratory fitness^49^ and increasing diversity after smoking cessation^50^. Hence, all of the above mentioned factors are potential confounders of the gut microbiota composition, which make direct comparison between the groups difficult.

However, the association of gut microbiota composition *and* KT ratio and neopterin in HIV-infected with T2D may represent a link between altered gut microbiota and activation of systemic inflammatory pathways. This could include the promotion of endothelial dysfunction as suggested by the independent association between KT ratio and ADMA, and this hypothesis should be further investigated in properly designed studies that also include longitudinal follow-up.

The main limitations of the present study include the cross-sectional design and a study cohort with several comorbidities and treatment modalities, which precludes any conclusions on causality. In particular, the lack of MSM status in the control group consisting of hospital staff, and the lack of nutritional data are major limitations, and comparison between groups should be interpreted with caution. Moreover, the number of individuals in each group was relatively low. Furthermore, the use of PICRUSt instead of full metagenomic shotgun sequencing represents a limitation for the assessment of functional aspects of the gut microbiota. The strengths of this exploratory study include the use of well-established protocols for gut microbiota sequencing and metabolomics, and the comparison of groups with HIV infection only, T2D only and both conditions.

In conclusion, our results may relate systemic inflammation and tryptophan catabolism to endothelial dysfunction, with a potentially detrimental interaction between HIV and T2D. The potential contribution of the gut microbiota and the impact for increased cardiovascular risk should be further explored in prospective studies powered for clinical end points and stratified for diabetes and MSM status.

## Electronic supplementary material

Supplementary Tables S1 and S2.
