# Supplementary material for: Impact of HIV and Type 2 diabetes on Gut Microbiota Diversity, Tryptophan Catabolism and Endothelial Dysfunction
Source: Sci Rep. 2018 Apr 30;8:6725. doi: 10.1038/s41598-018-25168-3 (PMC5928109; doi:10.1038/s41598-018-25168-3)
Supplement: Supplementary file 1 — Supplementary Tables S1 and S2. [file 41598_2018_25168_MOESM1_ESM.doc]

**Impact of HIV and Type 2 diabetes on Gut Microbiota Diversity, Tryptophan Catabolism and Endothelial Dysfunction**

Hedda Hoel, MD, Malene Hove-Skovsgaard, MD, Johannes R. Hov, MD PhD, Julie Christine Gaardbo, MD PhD, Kristian Holm, MSc, Martin Kummen, MD PhD, Knut Rudi, PhD, Felix Nwosu, MSc, Jørgen Valeur, MD PhD, Marco Gelpi, MD, Ingebjørg Seljeflot, PhD, Per Magne Ueland, MD PhD, Jan Gerstoft, MD DMSc, Henrik Ullum, MD PhD, Pål Aukrust, MD PhD, Susanne Dam Nielsen, MD DMSc, Marius Trøseid, MD PhD

**Supplementary Table S1.** Relative abundances (%) of significantly different taxa on order and genus level.

|  | **Control (n=24)** | **T2D (n=16)** | **HIV (n=23)** | **HIV+T2D (n=21)** | P* |
| --- | --- | --- | --- | --- | --- |
| **Order level** |  |  |  |  |  |
| Synergistales | 0.001 | 0.0007 | 0.007 | 0.02 | 0.010 |
| Actinomycetales | 0.01 | 0.03 | 0.01 | 0.16 | 0.012 |
| Bacillales | 0.04 | 0.01 | 0.004 | 0.02 | 0.020 |
| SHA-98 | 0.004 | 0.008 | 0.01 | 0.004 | 0.028 |
| Lactobacillales | 0.36 | 0.08 ^d^ | 0.18 ^d^ | 0.92 | 0.032 |
| Enterobacteriales | 2.3 | 3.5 | 1.8 | 6.3 | 0.050 |
| **Genus level** |  |  |  |  |  |
| *Lachnospira* | 0.96 ^d^ | 0.48 | 0.61 ^d^ | 0.28 | 0.011 |
| *Lachnobacterium* | 0.61 ^b,d^ | 0.42 | 0.44 | 0.32 | 0.033 |
| *Anaerostipes* | 0.28 ^d^ | 0.23 | 0.29 ^d^ | 0.07 | 0.002 |
| *Rothia* | 0.003 ^d^ | 0.003 ^d^ | 0.002 ^d^ | 0.1 | 0.013 |
| *Actinomyces* | 0.005 ^d^ | 0.007 | 0.003 ^d^ | 0.04 | 0.020 |
| *Eubacterium* | 0 ^c,d^ | 0 ^d^ | 0.001 ^d^ | 0.02 | 0.003 |
| *Alloscardovia* | 0.00004 ^d^ | 0.0001 | 0 ^d^ | 0.002 | 0.003 |
| *Acidaminococcus* | 0.18 ^c^ | 0.17 | 0.09 ^d^ | 0.43 | 0.020 |
| *Caulobacter* | 0 | 0 | 0 | 0.0009 | 0.027 |
| *Erysipelotrichacea-RFN20* | 0 | 0.0006 | 0.00005 | 0 | 0.030 |
| *Adlercreutzia* | 0.02 ^d^ | 0.03 | 0.03 ^d^ | 0.01 | 0.033 |
| *Streptococcus* | 0.24 | 0.06 ^d^ | 0.11^d^ | 0.71 | 0.044 |

P-value refers to Kruskal Wallis analysis. b, c, d refers to Mann Whitney U test t-test; b: p<0.05 vs. T2D, c: p<0.05 vs. HIV, d: p< 0.05 vs. HIV+T2D. *None of the p-values were significant (<0.05) after adjustment for multiple comparisons.

**Supplementary Table S2.** Bacterial taxa contributing to the rate limiting step for converting tryptophan to kynurenine, Indoleamine 2,3-oxygenase (IDO).

| **Bacterial taxa** | **Contribution (%)** |
| --- | --- |
| **Phylum level**  Proteobacteria | 81 |
| Actinobacteria | 12 |
| Firmicutes | 4.8 |
| Bacteroidetes | 1.2 |
| **Genus level** |  |
| *Comamonas* | 20 |
| *Delftia* | 7.2 |
| *Burkholderia* | 5.4 |
| *Micrococcus* | 4.5 |
| *Pseudomonas* | 4.2 |
| *Caulobacter* | 3.6 |
| *Sphingopyxis* | 3.6 |
| *Janthinobacterium* | 2.4 |
| *Alloscardovia* | 2.4 |
| *Bacillus* | 2.2 |
| *Phenylobacterium* | 1.8 |
| *Rhodococcus* | 1.2 |
| *Acidovorax* | 1.2 |
| *Exiguobacterium* | 1.2 |
| *Rhodoplanes* | 0.9 |

Bacterial contribution was calculated by the following formula: (abundance of OTU/number of 16s copies in OTU) x number of gene copies for the KEGG-pathway (K00453, EC: 1.13.11.11), downloaded at: <http://www.genome.jp/dbget-bin/www_bget?K00453>
